# Modeling the joint effects of adolescent and adult PrEP for sexual minority males in the United States

**DOI:** 10.1371/journal.pone.0217315

**Published:** 2019-05-22

**Authors:** Deven T. Hamilton, Eli S. Rosenberg, Samuel M. Jenness, Patrick S. Sullivan, Li Yan Wang, Richard L. Dunville, Lisa C. Barrios, Maria Aslam, Steven M. Goodreau

**Affiliations:** 1 Center for Studies in Demography and Ecology, University of Washington, Seattle, Washington, United States of America; 2 Department of Epidemiology and Biostatistics, University at Albany School of Public Health, State University of New York, Rensselaer, New York, United States of America; 3 Department of Epidemiology, Emory University, Atlanta, Georgia, United States of America; 4 Department of Global Health, Emory University, Atlanta, Georgia, United States of America; 5 Division of Adolescent and School Health, Centers for Disease Control and Prevention, Atlanta, Georgia, United States of America; 6 Program and Performance Improvement Office National Center for HIV, Viral Hepatitis, STD, and TB Prevention, Centers for Disease Control and Prevention, Atlanta, Georgia, United States of America; 7 Department of Anthropology, University of Washington, Seattle, Washington, United States of America; Agencia de Salut Publica de Barcelona, SPAIN

## Abstract

**Background:**

Pre-exposure prophylaxis (PrEP) is an effective and safe intervention approved for use to prevent HIV transmission. PrEP scale-up strategies and clinical practice are currently being informed by modeling studies, which have estimated the impact of PrEP in adult and adolescent MSM populations separately. This partitioning may miss important effects or yield biased estimates by excluding dependencies between populations.

**Methods:**

We combined two published models of HIV transmission among adults and adolescent MSM. We simulated an HIV epidemic among MSM aged 13–39 without PrEP, with PrEP for adult MSM ages (19–39) and with the addition of PrEP for adolescents ages (16–18), comparing percent of incident infections averted (impact), the number of person-years on PrEP per infection averted (efficiency), and changes in prevalence.

**Results:**

PrEP use among eligible 19–39 year old MSM averted 29.0% of infections and reduced HIV prevalence from 23.2% to 17.0% over ten years in the population as a whole. Despite being ineligible for PrEP in this scenario, prevalence among sexually active 18 year-olds declined from 6.0% to 4.3% due to reduced transmissions across age cohorts. The addition of PrEP for adolescents ages 16–18 had a small impact on the overall epidemic, further reducing overall prevalence from 17.0% to 16.8%; however prevalence among the sexually active 18 year-olds further declined from 4.3% to 3.8%.

**Conclusions:**

PrEP use among adults may significantly reduce HIV prevalence among MSM and may also have significant downstream effects on HIV incidence among adolescents; PrEP targeting adolescents remains an important intervention for HIV prevention.

## Introduction

Preexposure prophylaxis (PrEP) is an effective and safe intervention to prevent human immunodeficiency virus (HIV) [1–4]. The FDA first approved Truvada (Emtricitabine and Tenofovir Disoproxil Fumarate) for HIV PrEP among adults in 2012 [5] and the CDC clinical practice guidelines, issued in 2014, indicate PrEP use for sexually-active adult MSM with substantial HIV acquisition risk [6]. The potential impact of these policies has been demonstrated through modeling, which found that their application within a population of 18–39 year-old MSM could avert a significant number of HIV infections—33% in their baseline scenario [7]. The potential impact of PrEP on new HIV infections has also been demonstrated empirically, e.g. a recent analysis of new diagnoses among all persons ≥ 13 years of age in the US found that PrEP uptake is associated with a reduction in new HIV infections at the state level [8].

Adolescent sexual minority males (ASMM) are also at substantial population risk for HIV acquisition [9–13]. The subsequent ATN113 safety and feasibility study among US ASMM aged 15–17 [14] found PrEP adherence was lower than for the young adult men who have sex with men (MSM) in the ATN110 trial [15]; only 41.6% of ASMM were highly adherent (≥4 doses/week) on average, but there were no safety concerns apart from weight loss [16]. CDC updated their clinical practice guidelines for adolescents to recommend that the risks and benefits of PrEP be considered in the context of local regulations [17] and based on ATN113 results, the FDA approved the use of PrEP for adolescents in 2018 [16]. The full impact of making PrEP available to adolescents will not be known for many years, but a recent modeling study demonstrated that, despite relatively low adherence, PrEP use among sexually-active 16–18-year-old ASMM in high-risk US settings could reduce HIV incidence in this population (29.9%–41.1%) with a number-needed-to-treat (NNT) between 28 and 39, suggesting that PrEP could be a cost-effective intervention in this population [18]. (Note that the use of the term “adolescent sexually minority males” in this work and beyond, rather than the more familiar “adolescent males who have sex with males”, is chosen to reflect the fact that a substantial proportion of this population has not yet initiated sex, but might still benefit from sexual health education and promotion services. Analyses that are limited to those who have initiated sex refer to “sexually active ASMM”).

The modeling studies cited above showed promising results among adult MSM [7] and ASMM [18], respectively. However, the treatment of these populations separately, both in these studies and in others [7, 18–22], ignores multiple dependencies between them, which may impact predictions of PrEP’s overall impact in at least five ways:

Models of adult MSM may overestimate the impact of adult PrEP on MSM because they often assume that entrants into the population are all HIV-negative. However, recent CDC surveillance data indicates there are >1300 new HIV diagnoses each year among ASMM (ages 15–19) whose primary risk category is sexual contact with another male [9]; with relatively low rates of HIV testing in this group, the number of incident and prevalent infections is likely much higher [9]. Moreover, studies of ASMM in large urban centers including Chicago, Atlanta and New York [10, 11, 23] have found substantial HIV incidence, and prevalence estimates among 18–22 year-old MSM were 11–14% across three National HIV Behavioral Surveillance rounds [12, 13]. Models of HIV among adult MSM are typically calibrated to match empirical estimates of prevalence within the adult MSM population. The combined effect of these assumptions is that modeled transmission rates among adult MSM must be higher than reality to account for individuals who contribute to adult HIV prevalence but were infected as ASMM. The overestimated transmission rates are then subjected to adult PrEP interventions, and the averted infections are attributed to adult PrEP.

Separate models ignore the potential impact of adult PrEP on younger age cohorts. Several studies have reported that intergenerational sex is a key risk factor for HIV acquisition in young MSM [24–27]. A recent phylogenetic analysis of HIV-1 *pol* sequences among 23,048 US MSM indicated that 36% of MSM 13–24 years had a potential transmission partner who was >5 years older [28]. Another analysis of 3,102 *pol* sequences found that, of 35 clusters that included at least one adolescent MSM, only one included exclusively adolescents, and the median age of non-adolescents in those clusters was 32 [29]. In one Atlanta-based behavioral study, young adult MSM aged 19–25 reported that 6.7% of their relationships were with ASMM under 19 [30]. Consequently, an adult PrEP program that reduces HIV prevalence among young adult MSM, would also reduce transmissions from that population to ASMM because prevalence among the former group would be lower. Any downstream infections between ASMM then would also be averted.

Separate models ignore the potential impact of ASMM PrEP on adult MSM[7, 19, 20]. ASMM contribute to infections among young adult MSM in two ways. First, young adult MSM may acquire an infection directly from ASMM via sexual contact [24–28]. Second, HIV-infected ASMM age into the young adult MSM population. Reduced prevalence among ASMM because of ASMM PrEP will reduce both pathways.

Models of ASMM PrEP may underestimate the impact of PrEP on ASMM[18, 22]. Existing models of PrEP in ASMM have modeled the acquisition of infections from young adult MSM via a constant force of infection [18, 22]. However, as described above, ASMM PrEP should reduce prevalence among young adult MSM as well. These reductions should then feed back to further reduce incidence among ASMM.

Models of ASMM PrEP may overestimate the impact of PrEP on ASMM. ASMM PrEP may only delay some infections into adulthood rather than averting them. This distinction cannot be observed in ASMM-only models, where all reductions in incidence among ASMM are classified as averted infections. Of course, delayed infections might also avert some secondary infections to early-life partners of those whose infections are delayed. A combined model should detect the extent to which incidence is merely delayed by looking for an upward shift in the mean age at acquisition. Note that this mechanism has countervailing effects to the previous one, making *a priori* predictions of overall impact difficult.

This study aims to capture the effects of these dependencies by modeling a combined ASMM/adult MSM population from adolescence into young [19–25] and middle [26–39] adulthood. We first consider the impact of PrEP interventions targeted to adult MSM aged 19–39, as adult-focused programs have been underway across the US for several years. We then focus on the combined impact of adult PrEP with PrEP targeting ASMM aged 16–18, which we expect to rise in coming years.

## Methods

We combined two published stochastic dynamic network models, one for ASMM, defined as ages 13–18 [18], and one for adult MSM, defined here as ages 19–39 [7]. In short, we modeled an open cohort of ~13,500 13–39-year-olds (500 per 1-year age). We modeled sexual relationship formation and dissolution; sexual behavior within relationships (anal sex acts, condom use, role selection); HIV testing; HIV treatment initiation, adherence and cessation; PrEP initiation, adherence and discontinuation; transmission; intrahost viral dynamics; and demographic change (entry, aging, death and exit). The partnership networks were modeled using separable temporal exponential random graph models [31, 32]. At each time step (one- week) new partnerships could form, old partnerships could dissolve, and individuals engaged in within partnerships sexual decision making and behaviors. The model was implemented using the EpiModel platform (www.epimodel.org) [33]. Additional model details are provided in the supplemental material (S1 Appendix); here we detail elements that differ from those already published elsewhere [7, 18]. All simulation code and analysis scripts are available online at https://github.com/statnet/PrEP-for-ASMM-and-adult-MSM.

ASMM entered the population at age 13 and were assigned an age between 13 and 18 at which they would self-identify as ASMM, becoming eligible for sexual debut, with probabilities of 0.44, 0.13, 0.12, 0.11, 0.10, and 0.10 for each age 13–18, respectively. Once self-identified as ASMM individuals, they could enter the pool of possible sexual partnerships immediately with probability 0.56; otherwise they had a 0.0034 probability of debut per time step. The probabilities were back calculated [18] from data on the age at which individuals first began to self-identify as gay/bisexual and the age of sexual debut [34]. The details of this back calculation are available in the supplemental material (S1 Appendix). The multi-step process was designed to match age-specific levels of sexual activity among ASMM, limiting most sexual activity by ASMM to 16-18-year-olds, as observed in our source data [34].

The types of partnerships among adult MSM (main, casual and one-time) were the same as in previous models [7]; partnerships between ASMM were also the same as in prior models [18], and came in only one type, given fewer and less detailed data sources. Relationships between ASMM and young adult MSM (19–25) were new, and parametrized from the Checking In survey [30] with support from a phylogenetic analysis [28]. We set 6.7% of relationships reported by young adult MSM (19–25) to be with ASMM <19 to match the former source, while excluding contact between ASMM and MSM >25. In order to keep the total partnership counts for young adult MSM consistent with previous models, their partnerships with ASMM were subtracted from the casual partnerships they would have had with each other in the original adult MSM-only model. For ASMM, these partnerships replace the exogenous force of infection from individuals outside the age range that the ASMM-only models included.

Because we divided our population of 19–39 year old MSM into younger (19–25) and older (26–39) adult MSM, we also included an adjustment to increase incidence among the younger group to reflect the larger number of new diagnoses among this age group [9]. We did so by weighting the probability of being in a casual partnership by age group, assigning one-third of casual partnerships to older adult MSM and two-thirds to young adult MSM. This adjustment increased incidence among the younger adults and, absent age-specific data on casual partnerships, retained the overall mean number of casual partnerships among all 19–39 year olds to be consistent with prior models and the empirical data on which those models were based [7].

We calibrated our model using approximate Bayesian computation (ABC) to estimate parameter values for the frequency of anal intercourse (AI) within casual relationships between two adult MSM, AI frequency within relationships between two ASMM or an ASMM and young adult MSM, and condom use probabilities among ASMM. Our calibration targets were ~7% overall HIV prevalence among sexually-active 18-year-old ASMM [35] and ~28.3% prevalence among adult MSM [11]. The ABC used uniform priors and generated a 16.9% daily probability of AI within casual relationships among adult MSM, a 24.1% daily probability of AI within an ASMM relationship and a 23% probability that an ASMM would use a condom during AI.

Our analysis included five scenarios. In the baseline counterfactual scenario none of the individuals received PrEP. In the second scenario (most closely representing current practice) only adult MSM were eligible to receive PrEP, with eligibility based on CDC guidelines [6, 7]. Consistent with prior work, we modeled 40% coverage among eligible adult MSM, with coverage defined as the proportion of adult MSM meeting PrEP eligibility criteria who were currently using PrEP. We included four PrEP adherence levels, corresponding to no measurable adherence, low (<2 pills/week), medium (2–3 pills/week) and high (4+ pills/week). The proportion of adult PrEP users at each adherence level were 21.1%, 7.0%, 10.0%, and 61.9%, respectively, drawn from an open-label PrEP demonstration project [3].

The three ASMM intervention scenarios included the same PrEP intervention among adult MSM as modeled in the first intervention scenario, in addition to a PrEP intervention for 16-18-year-old ASMM. Following our earlier model [18], we considered ASMM as eligible for PrEP if they were 16–18 and had initiated AI, with an average 6-month delay between AI initiation and PrEP initiation, reflecting the average interval expected when sexual debut occurs between annual healthcare visits. The three interventions scenarios reflected coverage of 10%, 20%, and 30% among those eligible. These values were chosen under the assumption that adult coverage will likely always be higher than adolescent coverage, while still providing a range large enough to identify trends. PrEP adherence among 16-18-year-old ASMM was 20.9%, 24.4%, 13.1%, and 41.6% for no measurable adherence, low, medium, and high adherence, respectively; these reflect adherence averaged across all visits (4–48 weeks) in ATN113 [14]. For both adult MSM and ASMM the per-act transmission probability was reduced by 0%, 31%, 81%, and 95% for each respective adherence level based on derivations from Grant et al. [36].

For each scenario we ran 100 simulations for 40 years. The long time horizon was selected specifically to determine if PrEP implementation among ASMM averted or just delayed HIV acquisition, which required observing the ASMM cohort outcomes through the entire age range. The extent of delay was measured by considering the average age of infection in each scenario. However, we report most results based on a 10-year intervention window, as it represents a more realistic timeline for the evaluation of rapidly evolving public health interventions. Key outcomes we report are the number of infections averted per 100K person years at risk (NIA), the percentage of infections averted (PIA), and the number-needed-to-treat to avert a single infection (NNT). We also report changes in prevalence over time. For each outcome we report the mean across the 100 simulations and the 95% credible intervals (CrI; the middle 95% of observed values from the simulations). When reporting on prevalence among ASMM, we specifically report prevalence among 18-year-old ASMM for two reasons: given few deaths in this young population it is an approximation of cumulative incidence, and for consistency with prior simulation studies evaluating ASMM PrEP.

## Results

In the baseline scenario (no PrEP), the prevalence of HIV in the sexually active population was 23.2% (95% CrI: 21.4%, 24.5%) with incidence of 322.7 (95% CrI: 292.8, 344.2) per 10K person years at risk over 10 years (Table 1). With the inclusion of adult PrEP per CDC guidelines at 40% coverage among eligible adult MSM, there were on average 3,760 (95% CrI 3,660, 3,839) adult MSM on PrEP at any time. Over 10 years, overall prevalence declined to 17.0% (95% CrI: 15.7%, 18.0%), a 26.7% reduction; incidence declined to 206.4 (95% CrI: 187.4, 220.7) per 10K person years at risk; and 693 (95% CrI: 574, 820) infections were averted per 100K person years at risk, a PIA of 29.0% (95% CrI: 24.0%, 34.4%). The NNT was 33 (95% CrI: 27, 40). The average age at HIV acquisition increased only slightly from 25.9 (s.d. = 6.2) to 26.0 (s.d. = 6.6).

**Table 1:** The percentage of infection averted, the number of infections averted per 100K person years at risk, the number needed to treat to avert a single infection, prevalence, incidence, person-years HIV positive, and age at infection over 10 years of a PrEP intervention for adult men who have sex with men (MSM) and adolescent sexual minority males (ASMM).

Outcome (Mean and 95% Credible Interval)	PrEP coverage: adult MSM / ASMM				
	0% / 0%	40% / 0%	40% / 10%	40% / 20%	40% / 30%
**Percent of infections averted**	NA	29.0% (24.0%, 34.4%)	28.9% (23.2%, 34.1%)	29.7% (23.4%, 33.9%)	29.9% (23.6%, 35.0%)
**Number of infections averted per 100K person years at risk**	NA	693 (574, 830)	692 (551, 815)	711 (559, 816)	716 (558, 842)
**Number needed to treat to avert a single infection**	NA	33 (27, 40)	34 (28, 42)	34 (29, 40)	34 (29, 43)
**Prevalence (population)**	23.2 (21.4, 24.5)	17.0 (15.7, 18.0)	17.0 (15.7, 18.2)	16.8 (15.4, 18.0)	16.8 (15.4, 17.9)
**Prevalence among 18 year-olds**	6.0 (4.0, 8.3)	4.3 (2.3, 6.5)	4.2 (2.4, 5.9)	3.9 (1.7, 5.6)	3.8 (2.0, 6.0)
**Incidence per 10k person years**	323 (293, 344)	206 (187, 221)	206 (186, 223)	203 (184, 219)	203 (186, 218)
**Person years HIV+ per 10K person years**	2642 (2465, 2739)	2304 (2147, 2423)	2311 (2151, 2427)	2293 (2123, 2423)	2295 (2151, 2413)
Average age at infection*	25.9 (s.d. = 6.2)	26.0 (s.d. = 6.6)	26.0 (s.d. = 6.5)	26.1 (s.d. = 6.5)	26.2 (s.d. = 6.5)

*Note: Average age of infection is reported as a mean and standard deviation.

Over a longer time horizon of 40 years (Table 2), adult PrEP reduced overall prevalence to 7.4% (95% CrI: 6.0, 8.5) and incidence declined from 311.9 (95% CrI: 288.4, 328.1), to 133.1 (95% CrI: 117.7, 145.7). ASMM, who were not receiving PrEP under this scenario, also showed a reduction in prevalence among 18-year-old ASMM from 6.0% (95% CI: 4.0, 8.3) to 4.3% (95% CI: 2.30, 6.47) over ten years and a further reduction to 3.8% (95% CrI:2.0, 6.0) over 40 years (Tables 1 and 2).

**Table 2:** Prevalence, incidence, person years HIV positive and age at infection over 40 years of a PrEP intervention for adult men who have sex with men (MSM) and adolescent sexual minority males (ASMM).

Outcome (Means and 95% Credible Intervals)	PrEP coverage: adult MSM / ASMM				
	0% / 0%	40% / 0%	40% / 10%	40% / 20%	40% / 30%
**Prevalence (population)**	22.2 (20.1, 24.0)	7.4 (6.0, 8.5)	7.2 (5.4, 8.6)	7.0 (5.8, 8.0)	6.9 (5.5, 7.9)
**Prevalence among 18 year-olds**	6.7 (3.9, 9.1)	2.1 (0.4, 4.0)	1.9 (0.6, 3.6)	1.6 (0.4, 3.0)	1.5 (0.4, 3.0)
**Incidence per 10k person years**	311.9 (288.4, 328.1)	133.1 (117.7, 145.7)	132.1 (115.4, 146.0)	129.2 (113.8, 141.8	127.9 (113.7, 140.0)
**Person years HIV+ per 10K person years**	2557 (2401, 2691)	1481 (1343, 1582)	1478 (1336, 1586)	1454 (1304, 1556)	1445 (1327, 1542)
Average age at infection (over years 35–39)*	26.1 (s.d. = 6.3)	27.0 (s.d. = 6.8)	27.0 (s.d. = 6.8)	27.2 (s.d. = 6.8)	27.3 (s.d. = 6.7)

*Note: Average age of infection is reported as a mean and standard deviation.

The addition of PrEP for sexually active 16–18 ASMM with 10% coverage counterintuitively resulted in an average of one fewer infection averted per 100K years at risk, with an NIA of 692 (95% CrI 551, 815) and consequently a 0.1% reduction in the PIA (Table 1). These differences with the adult-only PrEP scenario are well within the range of stochastic variation; indeed, at just 10% coverage there were, on average, only 92 ASMM on PrEP at any time (95% CrI 86, 97) in a population of 13,500. Increasing PrEP coverage among ASMM to 20% increased the PIA from 29.0% with just adult PrEP to 29.7% (95% CrI: 23.4%, 33.9%), reflecting an increase in the NIA from 693 (95% CrI: 574, 830) to 711 (95% CrI: 559, 816). The additional increase in coverage to 30% had an additive impact on both NIA and PIA, increasing the NIA to 716 (95% CrI: 558, 842) and the PIA to 29.9% (95% CrI: 23.6%, 35.0%). At 30% coverage the number of ASMM on PrEP averaged 272 (95% CrI 254, 288). The overall increases in the NIA and the PIA with the introduction of PrEP for ASMM were small relative to the increases observed with the introduction of PrEP for adult MSM; however, the NNT only increased from 33 to 34 when PrEP for ASMM was included. The small change in the NNT indicates that there is little decline in efficiency associated with adding PrEP for ASMM.

The addition of ASMM PrEP at 30% coverage over 10 years reduced the prevalence of HIV from 17.0% with just adult PrEP to 16.8% (95% CrI: 15.4%,17.9%) and reduced prevalence among ASMM an additional 10.3% from 4.3 (95% CrI: 2.3, 6.5) to 3.8 (95%CI 2.0, 6.0). The addition of PrEP for ASMM increased the mean age of infection an additional 2.5 months to 26.2 years (s.d. = 6.5). Over the longer 40-year time horizon the addition of ASMM PrEP reduced the overall prevalence from 7.4% (95% CrI: 6.0, 8.5) to 6.9% (95% CrI:5.5, 7.9), reduced incidence to 127.9 (95% CrI: 113.7, 140.0) and increased the average age at infection to 27.3 (s.d. = 6.7; Table 2).

## Discussion

This study models the impact of PrEP targeted at both adult MSM and ASMM on the HIV epidemic. We model the sexual life course from debut through the first half of adulthood to focus specifically on impacts of PrEP that may not be captured when adult and adolescent populations are modeled independently.

Consistent with other adult MSM models, we found that PrEP use among at-risk adult MSM may have a significant and positive impact on the HIV epidemic, lowering incidence and, in turn, lowering prevalence. We found that at 40% coverage, 29% of infections were averted, with an NNT of 33. This was somewhat lower effectiveness and efficiency than was reported by a model of adult-only MSM PrEP [7], where they found that 33% of infections were averted and the NNT was 25. Although the exact causes for this difference in scale are hard to prove given a number of differences between models, our findings do suggest that the impact of adult PrEP on reducing HIV infections among adult MSM may be overestimated when only adult MSM are considered. This pattern makes sense, since adolescents can contribute to both incidence and prevalence among young adult MSM, but do not receive direct protection via PrEP when it is only available for adults. The magnitude of the potential overestimation, however, does not appear to be sufficient to detract from the core conclusions in prior studies: the expansion of PrEP programs can prevent a significant number of new HIV infections.

Perhaps the most striking finding is the potential impact of adult PrEP on ASMM that has been missed by existing age-separated models. Even when ASMM are not specifically targeted for PrEP uptake, adult PrEP can be an effective intervention for ASMM, reducing prevalence in this group by as much as 29% over 10 years, preventing transmissions of HIV from young adult MSM to ASMM by disrupting an essential mechanism for the persistence of the epidemic. The impact of age-mixing on HIV acquisition risk, particularly among the young, has been demonstrated in numerous contexts and populations [24, 26–29, 37, 38]. It has also been shown that young people in age-discordant relationships are more likely to engage in higher-risk behavior [24, 39, 40]. Increasing PrEP use among young adults may complement existing risk-reduction strategies for youth in age-discordant relationships [38] by reducing the environmental conditions—high partner HIV prevalence—from which their risk accrues. These downstream benefits provide additional support for the continued expansion of PrEP among at-risk MSM.

The population-level impact of ASMM PrEP was substantially smaller than what was observed with adult PrEP. This is not surprising, given that the ASMM population is a smaller proportion of the overall population, and prevalence among ASMM is substantially lower. ASMM were just 22% of the population, only half were 16–18 years old, and fewer still had sexually debuted, resulting in just 272 ASMM on PrEP on average even at 30% coverage or an additional ~2% of the population. However, we did find that, over ten years, PrEP for ASMM reduced prevalence in the population as a whole from 17.0% to 16.8%. Some of that reduction came from declines in prevalence among MSM, a process missed in single population models; however, most of the impact was among ASMM, for whom ASMM PrEP reduced prevalence from 4.3 (95% CI: 2.3, 6.5) to 3.8 (95% CI: 2.0, 6.0). This change cannot be directly compared to ASMM-only models because here the ASMM PrEP intervention is not implemented in isolation. However, Goodreau et al. reported that ASMM PrEP at 30% coverage reduced prevalence among 18-year-old ASMM by 23.1%. The reduction observed here in the presence of adult PrEP was 10.3%, a smaller, but still substantial, reduction. In addition, the inclusion of PrEP for ASMM had almost no impact on the efficiency of PrEP as measured by NNT, since the number of cases and person-years on PrEP contributed by adult MSM dwarfed the contributions by ASMM.

These findings on their face could be taken to suggest that PrEP for ASMM may not be a worthwhile policy due to its small impact on the overall HIV epidemic. However, we would make three points in response. First, for those ASMM for whom an infection is averted, the benefits are substantial and life-altering. The very small increase in mean age of infection, coupled with the decline in incidence, indicates that ASMM PrEP is indeed reducing new infections in these models, and not simply pushing infections from adolescence to adulthood. Thus, the small population-level impact should not be taken as a recommendation against providers prescribing PrEP to adolescents. Second, the efficiency of the PrEP intervention was not significantly impacted by the inclusion of PrEP for ASMM. Thus, while the impact at the population level was modest, so too was the number of person-years on PrEP required. Third, PrEP programs targeting ASMM may not have a large impact on the overall HIV epidemic among persons 13–39 years due to the small number of sexually active 16–18 year-old ASMM relative to the size of the entire MSM population. However, such a targeted program would significantly reduce the number of new infections among ASMM while helping to achieve health equity for this key at-risk population.

PrEP programs for ASMM may also be an important mechanism for supporting PrEP program for adults. Adolescent engagement with PrEP may set a lifelong norm, leading to higher retention and adherence in adulthood and establishing strong prevention habits like PrEP prior to the highest risk years. If early engagement with PrEP facilitates development of norms for treatment-seeking and engagement with healthcare professionals more generally, PrEP for ASMM may also indirectly facilitate improvement at each step along the care cascade [41, 42].

Our results suggest that modeling HIV prevention interventions within artificially bounded age limits, without taking into account interactions with individuals outside the modeled age range, can result in missing important intervention outcomes. Here we only modeled the inclusion of adult and adolescent age groups among MSM but the implications are likely more general. Different risk groups including MSM, heterosexuals, and persons who inject drugs are also likely to interact in ways that alter the epidemic trajectories within each group. When possible, epidemic projection models should strive to incorporate additional segments of the population to more accurately reflect real world epidemic dynamics. This will become even more important as we approach the goal of HIV elimination and the direction of model predictions become more sensitive to smaller changes in the underlying transmission dynamics. As with all model construction, choosing when to add these additional interactions requires evaluating the trade-offs between tractability and realism in the context of model goals, and will not always be possible due to data, resource and computational limits.

Our research had several limitations, including the exclusion of groups other than ASMM and adult MSM in our modeled population, the importance of which was discussed above. In addition, the age mixing between ASMM and the young adult MSM was governed by age-specific entry into the population and age-dependent sexual debut rather than by explicit age-mixing parameters due to a lack of empirical data on age-mixing patterns in this population. Given the important role that relationships between ASMM and young adult MSM play in determining the magnitude of the epidemic among ASMM, additional data on these mixing patterns will be needed in order to more accurately represent this process. Lastly, our model was limited to HIV. There is evidence that there are interactions between HIV and other STI [43] impacting both susceptibility [44, 45] and transmissibility [46], and that PrEP use could lead to higher STI incidence due to behavioral risk compensation; MSM who initiate PrEP may reduce their use of other disease prevention strategies [47]. This could lead to additional individual and public health burden from STIs directly, and might in turn reduce the overall impact of PrEP as increased STI prevalence could facilitate increased HIV incidence. However, at least one study has suggested that the inclusion of regular screening and treatment of STI along with the recommended HIV testing for PrEP users may actually be an effective intervention for reducing both HIV and STI, as it increases the frequency of testing and reduces the time from infection to treatment [48].

For the past four decades, each cohort of ASMM has faced a different HIV epidemic, and has had different sets of prevention modalities available to them at different times in their lives. The current generation of ASMM is the first to come of age with PrEP as a viable option for HIV prevention from the start, and as a consequence, there is the real possibility of preventing the epidemic from establishing itself in force among younger cohorts. Our work shows that the continued expansion of PrEP for adults in conjunction with PrEP programs focused specifically on ASMM can reduce the prevalence of HIV by a third among ASMM in just 10 years and by 75% over 40 years without any additional changes in treatment or prevention, bringing us much closer to the goal of an AIDS-free generation.

## Supporting information

S1 Appendix Supplementary technical appendix. (DOCX)
