# Supplementary material for: Modeling the joint effects of adolescent and adult PrEP for sexual minority males in the United States
Source: PLoS One. 2019 May 22;14(5):e0217315. doi: 10.1371/journal.pone.0217315 (PMC6530873; doi:10.1371/journal.pone.0217315)
Supplement: S1 Appendix — (DOCX) [file pone.0217315.s001.docx]

Modeling the joint effects of adolescent and adult PrEP for sexual minority males in the US

Authors: D. T. Hamilton, MPH PhD^1^, E. S. Rosenberg, PhD^2^, S. M. Jenness, PhD^3^, P. S. Sullivan, DVM PhD^3,4^, L. Wang, MBA MA^5^, R. L. Dunville, MPH^5^, L. C. Barrios, DrPH^5^, M. Aslam, PhD^6^, S. M. Goodreau, PhD^1,7^

^1^ Center for Studies in Demography and Ecology, University of Washington, Seattle, WA

^2^ Department of Epidemiology and Biostatistics, University at Albany School of Public Health, State University of New York, Rensselaer, NY

^3^ Department of Epidemiology, Emory University, Atlanta, GA

^4^ Department of Global Health, Emory University, Atlanta, GA

^5^ Division of Adolescent and School Health, Centers for Disease Control and Prevention, Atlanta, GA

^6^ Program and Performance Improvement Office National Center for HIV, Viral Hepatitis, STD, and TB Prevention, Centers for Disease Control and Prevention, Atlanta, GA

^7^ Department of Anthropology, University of Washington, Seattle, WA

***Supplementary Technical Appendix***

**TABLE OF CONTENTS**

1 INTRODUCTION 2

1.1 Model Framework 2

1.2 Model Software 3

2 EMPIRICAL DATA 3

3 NETWORKS OF SEXUAL PARTNERSHIPS 4

3.1 Conceptual Representation of Sexual Networks 4

3.2 Statistical Representation of Sexual Networks 9

4 BEHAVIOR WITHIN SEXUAL PARTNERSHIPS 13

4.1 Disclosure 13

4.2 Number of AI Acts 13

4.3 Condom use 14

4.4 Sexual role 15

5 DEMOGRAPHY 15

5.1 Entry at Sexual Onset 15

5.2 Initialization of Attributes 8

5.3 Exits from the Network 18

5.4 Aging 19

6 INTRAHOST EPIDEMIOLOGY 19

7 CLINICAL EPIDEMIOLOGY 20

7.1 HIV Diagnostic Testing 20

7.2 Antiretroviral Therapy (ART) Initiation 21

7.3 ART Adherence and Viral Suppression 22

7.4 Disease Progression and Mortality after ART Initiation 22

8 INTERHOST EPIDEMIOLOGY 23

8.1 Disease-Discordant Dyads 23

8.2 Per-Act HIV Transmission Probability 23

9 PRE-EXPOSURE PROPHYLAXIS (PrEP) 24

9.1 PrEP Indications 24

9.2 PrEP Uptake and Monitoring 25

9.3 Adherence and Impact on HIV Transmission 25

10 SIMULATION METHODS 26

10.1 Model Calibration 26

10.2 Intervention Simulations 26

11 REFERENCES 27

# 1 INTRODUCTION

This supplementary technical appendix describes the mathematical model structure, parameterization, and statistical analysis of the accompanying paper in further detail.

## 1.1 Model Framework

The mathematical models for HIV transmission dynamics presented in this study are agent-based microsimulation models in which uniquely identifiable sexual partnership dyads were simulated and tracked over time. This partnership structure is represented through the use of separable temporal exponential-family random graph models (STERGMs), described in Section 3. On top of this dynamic network simulation, the larger epidemic model represents demography (entries, exits, and aging), interhost epidemiology (disease transmission), intrahost epidemiology (disease progression), and clinical epidemiology (disease diagnosis and treatment). Individual attributes related to these processes are stored and updated in discrete time over the course of each epidemic simulation.

The modeling methods presented here depend upon and extend the *EpiModel* software to incorporate HIV-specific epidemiology. The HIV extensions for men who have sex with men (MSM) were originally developed by Goodreau et al. for use in prior modeling studies of MSM in the United States and South America (1-3), and subsequently in a research project investigating the causes and consequences of racial disparities in HIV incidence among young MSM in the US (4), the evaluation of the CDC pre-exposure prophylaxis (PrEP) guidelines (5) and other research. The extensions specific to adolescent sexual minority males (ASMM) were originally developed for use in prior modeling studies evaluating the use of PrEP among ASMM (6).

The model algorithms and methods presented here merge these prior MSM HIV transmission models to investigate PrEP use among both adolescent and adults in a unified population that includes self-identification as an ASMM, age at first sexual intercourse, and aging into early and middle adulthood. This work is part of a collaborative modeling effort (the Coalition for Applied Modeling for Prevention) between Emory University, the University of Washington, University at Albany, and the Centers for Disease Control and Prevention [http://emorycamp.org/].

## 1.2 Model Software

The models in this study were programmed in the R and C++ software languages using the *EpiModel* [http://epimodel.org/] software platform for epidemic modeling. The *EpiModel* platform supports the simulation of complex network-based mathematical models of infectious diseases, with a primary focus on HIV and other sexually transmitted infections (STIs). *EpiModel* depends on *Statnet* [http://statnet.org/], a suite of software in R for the representation, visualization, and statistical analysis of complex network data (7).

Simulations were performed on the Hyak high-performance computing (HPC) system at the University of Washington. This 11,000-core HPC allowed execution of multiple simulations in parallel to reduce the overall computation time.

# 2 EMPIRICAL DATA

The parameters for the behavioral modules for the MSM within the larger epidemic model are drawn from a previously published study (5). Data descriptions and analysis can be found in the cited manuscript and the associated supplemental material. Briefly, the authors used two studies of HIV/STI disparities in black and white non-Hispanic MSM, conducted from 2010–2014 in Atlanta, Georgia. The **Involvement Study** was a prospective HIV incidence cohort of 803 MSM and the **MAN Project** was a cross-sectional chain-referral sexual network study of 314 MSM. Both samples were recruited contemporaneously using venue-time-space sampling, using a modified frame from the 2008 cycle of the National HIV Behavioral Surveillance system. Study participants completed common self-administered computer-based questionnaire modules that assessed demographics, prevention behaviors, and a detailed dyadic (partnership) section that collected demographic, behavioral, and structural (partnership duration and sequence) data.

The parameters that were specific to the ASMM in the modeled population were drawn from the literature and they are noted within each section below.

Due to the broad focus of this modeling paper, we did not explicitly model race/ethnicity either in the input parameters or as output statistics.

# 3 NETWORKS OF SEXUAL PARTNERSHIPS

We modeled networks of three interacting types of sexual relations among adult MSM: main partnerships, casual (but persistent) partnerships, and one-time anal intercourse (AI) contacts. For the ASMM we modeled only a single relationship type that we will refer to simply as ASMM relationships. We first describe the methods conceptually, including the parameters used to guide the model and their derivation (Section 3.1), and then present the formal statistical modeling methods (Section 3.2). Consistent with our parameter derivations, all relationships are defined as those in which AI is expected to occur at least once.

## 3.1 Conceptual Representation of Sexual Networks

Our modeling methods aim to preserve certain features of the cross-sectional and dynamic network structure as reported in behavioral studies, while also allowing for mean relational durations to be targeted to those reported for different groups and relational types. These methods do so all within the context of changing population size (due to births, deaths, arrivals and departures from the population) and changing composition by attributes such as age and disease status.

The network features that we aim to preserve are as follows, with the parameters for each described in turn:

For the adult MSM

- The proportion of men in any given combination of main and casual partnerships (for example, in 1 main and 0 casual partnerships) at any time point.
- The expected number of one-time contacts per time step had by men in each main-casual combination.
- The proportion of relationships reported by young adult MSM that are with ASMM < 19 years of age.
- Variation across men in the numbers of one-time contacts.
- Age mixing within each of the different relational types.
- Prohibitions against partnering for two men who are both exclusively insertive or exclusively receptive.

For the ASMM

- The proportion of ASMM in any relationship that includes AI at any point in time.
- Prohibition against partnering for two ASMM who are both exclusively insertive or exclusively receptive.
- Prohibition against partnering with an MSM > 25 years of age.

*3.1.1 Number of Ongoing Partnerships (Main / Casual / ASMM)*

Ongoing partnerships (whether main or casual) were defined from the combined dyadic dataset as those in which sex had already occurred more than once, and in which the respondent anticipated having sex again. Within this set, partnerships were defined as main if the respondent indicated that it was someone they “felt committed to above all others” or that they considered the person their “primary sex partner”; if neither of these conditions held, the partner was defined as casual. This yielded the following proportions of men with a given number of main and casual relationships at a point in time (i.e., the expected *momentary degree distribution*):

|  | *0 Casual* | *1 Casual* | *2 Casual* |
| --- | --- | --- | --- |
| *0 Main* | 47.1% | 16.7% | 7.4% |
| *1 Main* | 22.0% | 4.7% | 2.1% |

This is the momentary degree distribution used in multiple prior studies of the adult MSM population. However, for this study we include relationships between ASMM and YAMSM (19-25) based on data from the Checking In survey (8) with support from Whiteside et al. 2015 (9). We parametrized our model to match the empirical data, which indicated that 6.7% of relationships reported by YAMSM (19-25) were with ASMM younger than 19, while excluding contact between ASMM and MSM older than 25. In order to keep the number of partnerships for the YAMSM consistent with previous studies, their partnerships with ASMM were subtracted from the casual partnership counts they would have had with each other in the original model that was limited to 18-39 year old MSM. Subtracting 6.7% of the casual partnerships from just the YAMSM changed the overall expected momentary degree distribution for all adult MSM 19-35 that was used as the target momentary degree distribution in the simulation to:

|  | *0 Casual* | *1 Casual* | *2 Casual* |
| --- | --- | --- | --- |
| *0 Main* | 48.2% | 15.6% | 7.4% |
| *1 Main* | 22.0% | 4.7% | 2.1% |

For the ASMM, the momentary degree distribution was based on reports from Halkitis 2013 (10) that 27% of sexually active ASMM are in a male-male relationships at any given time. The 0.27 mean degree set the target mean degree for sexually active ASMM. In this model not all ASMM are sexually active (see section 5.1). Thus, at the population level, including the proportion of ASMM that are not sexually active, the adjusted mean degree is 0.162. In order to account for heterogeneity in sexual activity, we model five risk groups. For these, the propensity to form partnerships was weighted by the quintile distribution of the number of anal sex partners reported in the last year by ASMM in the American Men’s Internet Survey, or AMIS (11). The parameters for momentary degree among sexually active ASMM guiding this model are:

| **Quintile** | **Mean momentary degree** |
| --- | --- |
| Lowest quintile | 0.054 |
| Second quintile | 0.106 |
| Third quintile | 0.161 |
| Fourth quintile | 0.247 |
| Highest quintile | 0.782 |
| Mean | 0.270 |

*3.1.2 Expected Number of One-Time AI Contacts, by Main/Casual Degree*

Respondents in the combined dyadic dataset were asked whether they had had sex with each partner once or more than once; the former response led to the contact being defined as one-time. These contacts cannot be analyzed in terms of momentary degree distributions, since none are ongoing at the point of interview, by definition. Instead, we turn the observed frequencies into expected rates of one-time contacts per time step for men under different conditions. One of the sources of heterogeneity in men’s propensity for one-time AI contacts is their current relationship status. The expected numbers are given by:

|  | *0 Casual* | *1 Casual* | *2 Casual* |
| --- | --- | --- | --- |
| *0 Main* | 0.065 | 0.087 | 0.086 |
| *1 Main* | 0.056 | 0.055 | 0.055 |

ASMM did not have one time contacts in the simulation.

*3.1.3 Heterogeneity in the One-Time Contact Rate* for adult MSM

In addition to differences by relational status, adult men also have underlying fixed heterogeneities in their propensity to engage in one-time AI. The distribution of one-time contacts was divided into quintiles, within which the expected values of one-time AI per time step are:

| **Quintile** | **Value** |
| --- | --- |
| Lowest quintile | 0.000 |
| Second quintile | 0.007 |
| Third quintile | 0.038 |
| Fourth quintile | 0.071 |
| Highest quintile | 0.221 |

Men are assigned a quintile upon entry into the population, which remains fixed. Any individual man’s propensity for AI is determined as a combination of their quintile and their current main/casual partnership counts. Our statistical methods (described below) translate both propensities into conditional log-odds, allowing for their combination. Note that the means of the columns in the quintile table equal the means of the values in Section 3.1.2 weighted by the proportions in Section 3.1.1. These reflect the overall expected value across all men for one-time AI acts per time step.

*3.1.4 Age Mixing*

Respondents in the studies of adult MSM also reported on the estimated age of each partner. We model age mixing within a given relational type using a single parameter for each, the expected mean difference in square root of the ages of men in a relationship, consistent with previous work (1, 3, 12). For instance, a relationship between a 23-year-old and a 28-year-old would represent $\left| \sqrt{23}-\sqrt{28} \right|$ = 0.496.

|  | **Value** |
| --- | --- |
| Main partnerships | 0.464 |
| Casual partnerships | 0.586 |
| One-time contacts | 0.544 |

Data on age mixing among ASMM was not available so we did not include an explicit parameter in the model to govern age mixing among ASMM. As with our previous models (6) (13) sexual activity by ASMM concentrated among the older ASMM through a process of self-identifying as an ASMM and sexual debut (see section 5.1).

*3.1.5 Mixing by Sexual Role*

We assign the adult MSM and ASMM a fixed sexual role preference (exclusively insertive, exclusively receptive, versatile). The model then includes an absolute prohibition, such that two exclusively insertive men cannot partner, nor can two exclusively receptive men. For the adult men the role probabilities were calculated based on aggregated reports of roles at last sex for each of the last 5 (Involvement) or 10 (MAN Project) partners. Those who had engaged in one role across all of those acts were deemed to be exclusively receptive or insertive, and those who had engaged in at least one act of each were deemed to be versatile.

|  | **Probability** |
| --- | --- |
| Exclusively insertive | 24.2% |
| Versatile | 43.7% |
| Exclusively receptive | 32.1% |

For the ASMM, the role proportions were based on estimates from the American Men’s Internet Survey (AMIS), and as with the adult MSM, partnering between two ASMM who were exclusively receptive or exclusively insertive was forbidden in the relational model.

|  | **Probability** |
| --- | --- |
| Exclusively insertive | 14.5% |
| Versatile | 57.3% |
| Exclusively receptive | 28.2% |

Because the estimates of the population proportions for each role class were different for the adult MSM and the ASMM, a small fraction of the ASMM were reclassified when they reached age 19 and thereby transitioned from being an ASMM to being a YAMSM. All exclusively insertive and exclusively receptive ASMM retained their role as they aged into adulthood while 13.6% of the ASMM who were classified as versatile as ASMM were reclassified as either exclusively insertive or exclusively receptive to match the role proportions among the adult MSM. The ASMM for whom we changed the role designation were selected from among the ASMM not in a relationship as the time of the reclassification to avoid inadvertently creating partnerships between two exclusively receptive or two exclusively insertive individuals.

*3.1.6 Partnership Durations*

We model relational dissolution as a memoryless process with a single parameter per relational type. This implies an exponential distribution for relational durations within each category. As detailed in previous work, for memoryless processes, the expected age of an extant relationship at any moment in time matches the expected uncensored duration of relationships, given the balancing effects of right-censoring and length bias for this distribution. To derive our values, we take the median of the observed distribution and then calculate the mean for the exponential distribution with that median. For the adult MSM, the duration was calculated as the difference between first and last sex date for each dyad the ego reported sex with more than once in the interval. For the ASMM, we examined data from AMIS (11), limiting to respondents 18 or younger. The resulting expected relational durations were:

|  | **Duration** |
| --- | --- |
| Main partnerships | 407 days |
| Casual partnerships | 166 days |
| ASMM partnership | 20 days |

## 3.2 Statistical Representation of Sexual Networks

Exponential-family random graph models (ERGMs) and their dynamic extension separable temporal ERGMs (STERGMs) provide a foundation for statistically principled simulation of local and global network structure given a set of target statistics from empirical data. Main and casual relationships were modeled using STERGMs (14), since they persist for multiple time steps. One-time contacts, on the other hand, were modeled using cross-sectional ERGMs (15). Formally, our statistical models for relational dynamics can be represented as five equations for the conditional log odds (logits) of relational formation and persistence at time *t* (for main and casual relationships) or for relational existence at time *t* (for one-time contacts):

$logit\left( P\left( Y_{ij,t}=1 | Y_{ij,t-1}=0\text{, }Y_{ij,t}^{C} \right) \right)\text{ = }{\theta_{m}^{+}}^{'}\partial\left( g_{m}^{+}\left( y \right) \right)$ Main partnership formation

$logit\left( P\left( Y_{ij,t}=1 | Y_{ij,t-1}=0\text{, }Y_{ij,t}^{C} \right) \right)\text{ = }{\theta_{c}^{+}}^{'}\partial\left( g_{c}^{+}\left( y \right) \right)$ Casual partnership formation

$logit\left( P\left( Y_{ij,t}=1 | Y_{ij,t-1}=0\text{, }Y_{ij,t}^{C} \right) \right)\text{ = }{\theta_{a}^{+}}^{'}\partial\left( g_{a}^{+}\left( y \right) \right)$ ASMM partnership formation

$logit\left( P\left( Y_{ij,t}=1 | Y_{ij,t-1}=1\text{, }Y_{ij,t}^{C} \right) \right)\text{ = }{\theta_{m}^{-}}^{'}\partial\left( g_{m}^{-}\left( y \right) \right)$ Main partnership persistence

$logit\left( P\left( Y_{ij,t}=1 | Y_{ij,t-1}=1\text{, }Y_{ij,t}^{C} \right) \right)\text{ = }{\theta_{c}^{-}}^{'}\partial\left( g_{c}^{-}\left( y \right) \right)$ Casual partnership persistence

$logit\left( P\left( Y_{ij,t}=1 | Y_{ij,t-1}=1\text{, }Y_{ij,t}^{C} \right) \right)\text{ = }{\theta_{a}^{-}}^{'}\partial\left( g_{a}^{-}\left( y \right) \right)$ ASMM partnership persistence

$logit\left( P\left( Y_{ij,t}=1 | Y_{ij,t}^{C} \right) \right)\text{ = }{\theta_{o}}^{'}\partial\left( g_{o}\left( y \right) \right)$ One-time contact existence

where:

- $Y_{ij,t}$ = the relational status of persons *i* and *j* at time *t* (1 = in relationship/contact, 0 = not)
- $Y_{ij,t}^{C}$ = the network complement of *i,j* at time *t*, i.e. all relations in the network other than *i,j*
- $g\left( y \right)$ = vector of network statistics in each model
- $\theta$ = vector of parameters in the formation model

For $g\left( y \right)$ and $\theta$, the superscript distinguishes the formation model (+), persistence model (-) and existence models (neither). The subscript indicates the main (m), casual (c), ASMM (a) and one-time (o) models.

The recursive dependence among the relationships renders the model impossible to evaluate using standard techniques; we use Markov chain Monte Carlo (MCMC) methods in order to obtain the maximum likelihood estimates for the $\boldsymbol{\theta}$ vectors given the $\boldsymbol{g}\left( \boldsymbol{y} \right)$ vectors.

Specific model statistics are listed below. Together these sets allow us to retain all of the network features listed in Section 3.1. it is important to note that, although the statistics are expressed here in terms of number of relationships and enter into the estimation model in this form, the simulation model is then parametrized using the resulting $\theta$ coefficients. This means that, as population size and composition changes, it is not the absolute number of relationships of different kinds that will be preserved, but the relative numbers (e.g., the mean number of relationships per person). Similar conversions hold for the other statistics (e.g., the mean age difference per relationship is preserved, not the sum across all relationships).

Main partner formation model statistics: $g_{m}^{+}\left( y \right)$ vector:

- $g_{m1}^{+}\left( y \right)$ = number of main partnerships
- $g_{m2}^{+}\left( y \right)$ = number of men with 2+ main partners
- $g_{m3}^{+}\left( y \right)$ = number of main partnerships for men with 1 casual partner
- $g_{m4}^{+}\left( y \right)$ = number of main partnerships for men with 2 casual partners
- $g_{m5}^{+}\left( y \right)$ = sum of the absolute difference in the square root of partners’ ages across main partnerships
- $g_{m6}^{+}\left( y \right)$ = number of main partnerships between men who were both exclusively insertive
- $g_{m7}^{+}\left( y \right)$ = number of main partnerships between men who were both exclusively receptive
- $g_{m8}^{+}\left( y \right)$ = number of main partnerships for ASMM

There are structural zeros as coefficient constraints for the terms $g_{m2}^{+}\left( y \right)$, $g_{m6}^{+}\left( y \right)$, $g_{m7}^{+}\left( y \right)$, $g_{m8}^{+}\left( y \right)$. This means that the logit values for their coefficients are set to negative infinity to ensure that no partnerships of these types occur.

Main partner persistence model terms: $g_{m}^{-}\left( y \right)$ vector:

- $g_{m1}^{-}\left( y \right)$ = number of main partnerships

Casual partner formation model terms: $g_{c}^{+}\left( y \right)$ vector:

- $g_{c1}^{+}\left( y \right)$ = number of casual partnerships
- $g_{c2}^{+}\left( y \right)$ = number of casual partnerships for men with 1 main partner
- $g_{c3}^{+}\left( y \right)$ = number of men with 2 casual partners
- $g_{c4}^{+}\left( y \right)$ = number of men with 3+ casual partners
- $g_{c5}^{+}\left( y \right)$ = sum of the absolute difference in the square root of partners’ ages across casual partnerships
- $g_{c6}^{+}\left( y \right)$ = number of casual partnerships between men who were both exclusively insertive
- $g_{c7}^{+}\left( y \right)$ = number of casual partnerships between men who were both exclusively receptive
- $g_{c8}^{+}\left( y \right)$ = number of casual partnerships for ASMM
- $g_{c9}^{+}\left( y \right)$ = number of casual partnerships for adult MSM older than 25

There are structural zeros as coefficient constraints for the terms $g_{c4}^{+}\left( y \right)$, $g_{c6}^{+}\left( y \right)$, $g_{c7}^{+}\left( y \right)$ $g_{c8}^{+}\left( y \right)$. This means that the logit values for their coefficients are set to negative infinity to ensure that no partnerships of these types occur.

Casual partner persistence model terms:$g_{c}^{-}\left( y \right)$ vector:

- $g_{c1}^{-}\left( y \right)$ = number of casual partnerships

ASMM partner formation model terms: $g_{a}^{+}\left( y \right)$ vector:

- $g_{a1}^{+}\left( y \right)$ = number of ASMM partnerships
- $g_{a2}^{+}\left( y \right)$ = the number of ASMM partnerships for each risk group quintile
- $g_{a3}^{+}\left( y \right)$ = number of ASMM partnerships with an YAMSM
- $g_{a4}^{+}\left( y \right)$ = number of ASMM partnerships with an MSM > 25 years of age
- $g_{a5}^{+}\left( y \right)$ = number of ASMM partnerships between two YAMSM
- $g_{a6}^{+}\left( y \right)$ = number of ASMM partnerships for ASMM that have not become sexually active
- $g_{a7}^{+}\left( y \right)$ = number of casual partnerships between men who were both exclusively insertive

There are structural zeros as coefficient constraints for the terms $g_{m4}^{+}\left( y \right)$, $g_{m5}^{+}\left( y \right)$, $g_{m6}^{+}\left( y \right)$, $g_{m7}^{+}\left( y \right)$. This means that the logit values for their coefficients are set to negative infinity to ensure that no partnerships of these types occur.

ASMM partner persistence model terms:$g_{a}^{-}\left( y \right)$ vector:

- $g_{a1}^{-}\left( y \right)$ = number of casual partnerships

One-time contact existence model terms:$g_{o}\left( y \right)$ vector:

- $g_{o1}\left( y \right)$ = number of one-time contacts
- $g_{o2}\left( y \right)$ = total # of one-time contacts for men with 0 main and 1 casual partnership
- $g_{o3}\left( y \right)$ = total # of one-time contacts for men with 0 main and 2 casual partnerships
- $g_{o4}\left( y \right)$ = total # of one-time contacts for men with 1 main and 0 casual partnerships
- $g_{o5}\left( y \right)$ = total # of one-time contacts for men with 1 main and 1 casual partnership
- $g_{o6}\left( y \right)$ = total # of one-time contacts for men with 1 main and 2 casual partnerships
- $g_{o7}\left( y \right)$ = total # of one-time contacts for men in risk quintile 1
- $g_{o8}\left( y \right)$ = total # of one-time contacts for men in risk quintile 2
- $g_{o9}\left( y \right)$ = total # of one-time contacts for men in risk quintile 4
- $g_{o10}\left( y \right)$ = total # of one-time contacts for men in risk quintile 5
- $g_{o11}\left( y \right)$= sum of the absolute difference in the square root of partners’ ages across one-time contacts
- $g_{o12}^{+}\left( y \right)$ = number of one-time contacts between men who were both exclusively insertive
- $g_{o13}^{+}\left( y \right)$ = number of one-time contacts between men who were both exclusively receptive
- $g_{o14}^{+}\left( y \right)$ = number of one-time contacts with an ASMM

There are structural zeros as coefficient constraints for the terms $g_{o12}^{+}\left( y \right)$, $g_{o13}^{+}\left( y \right)$, $g_{o14}^{+}\left( y \right)$. This means that the logit values for their coefficients are set to negative infinity to ensure that no partnerships of these types occur.

Our method of converting the statistics laid out in Section 3.1 into our fully specified network models consists of the following steps:

1. Construct a cross-sectional network of 13,500 men (500 for each age 13 to 39) with no relationships.
2. Assign adult MSM and ASMM sexual roles based on frequencies listed in Section 3.1.6, as well as risk quintiles (20% of the men per quintile) used for one-time partnership propensities for adult MSM and ASMM partnership propensities for ASMM.
3. Calculate the target statistics (i.e., the expected count of each statistic at any given moment in time) associated with the terms in the formation model (for the main, casual and ASMM partnerships) and in the existence model (for one-time contacts).
4. Assign each node a place-holder main and casual degree (number of on-going partnerships) that is consistent with the degree matrices, and store these numbers as a nodal attribute. (Note: this does not actually require individuals to be paired up into the partnerships represented by those degrees).
5. For the main, casual and ASMM networks, use the mean relational durations to calculate the parameters of the persistence model, using closed-form solutions, given that the models are dyadic-independent (each relationship’s persistence probability is independent of all others).
6. For the main, casual and ASMM networks, estimate the coefficients for the formation model that represent the maximum likelihood estimates for the expected cross-sectional network structure.
7. For the one-off network, estimate the coefficients for the existence model that represent the maximum likelihood estimates for the expected cross-sectional network structure.

Steps 5–7 occur within the *Statnet* software, and use the ERGM and STERGM methods therein. They are made most efficient by the use of an approximation in Step 6 (16). During the subsequent model simulation, we use the method of Krivitsky et al. (17) to adjust the coefficient for the first term in each model at each time step, in order to preserve the same expected mean degree (relationships per person) over time in the face of changing network size and nodal composition. At all stages of the project, simulated partnership networks were checked to ensure that they indeed retained the expected cross-sectional structure and relational durations throughout the simulations.

# 4 BEHAVIOR WITHIN SEXUAL PARTNERSHIPS

We model four phenomena consecutively within relationships at each time step: HIV+ status disclosure, number of anal sex acts, condom use per sex act, and sexual role per sex act.

## 4.1 Disclosure

We model the process by which someone who knows he is HIV-positive discloses this fact to partners of all types. Disclosure affects subsequent decision-making around condom use. We do not explicitly model other forms of serostatus discussion, since our source data do not include these all. Our behavioral estimates for adult MSM in the absence of HIV+ disclosure are marginalized over those cases in which men disclose as concordant negative and do not discuss at all. Disclosure among adult MSM may occur at the point of a relation commencing (if HIV+ status is already known) or it may occur at the point of diagnosis, in the case of on-going relationships. In the former case, disclosure of HIV+ status was determined from the combined dyadic dataset using the HIV status of the respondent and their response to the question, “Did you and this partner share both of your HIV statuses before you first had sex?” In the latter case, we did not have data and assumed it to be universal. Disclosure estimates for ASMM were not available at the time this research was conducted so the ASMM only disclose if they are in an ongoing relationship at the time of diagnosis. Due to the relatively low testing rates among ASMM (see section 7.1), very few ASMM are likely to be diagnosed while in a partnership and disclose their status.

| Probability of Disclosure of HIV+ Status | Probability |
| --- | --- |
| to new main partner at outset of relationship | 78.7% |
| to new casual partner at outset of relationship | 67.8% |
| to one-time contact | 56.8% |
| to ongoing partner if diagnosis occurs during relationship | 100% |

##

## 4.2 Number of AI Acts

The number of anal sex acts per week for each ongoing relationship is determined from a Poisson draw, with mean specific to the relational type. For one-time contacts, the number is set deterministically to 1 for the time step in which it occurs.

| **AI Acts/Week/Partnership** | **Frequency** |
| --- | --- |
| Main partnerships | 1.54 |
| Casual partnerships | 0.96 |
| ASMM partnerships | 0.27 |

The rates for the main and casual partnerships among adult MSM were calculated based on the two Atlanta studies, derived from questions asking the number of coital acts per partnership during the recall periods (18) (8). These were then rescaled from the length of the recall period into the weekly rates listed in the table above. For the ASMM the baseline frequency of AI within ongoing relationships was derived from Rotheram-Borus et al. 1994 (19), who reported a mean of 3.5 acts of AI over a three month period, or 0.038 acts per day. AI frequency was one of the parameters adjusted using Approximate Bayesian Computation (ABC) to fit the HIV prevalence value chosen as our calibration parameter.

## 4.3 Condom use

In order to obtain condom use estimates for the adult MSM we conducted logistic regressions to identify the significant predictors of condom use within HIV-discordant relationships (whether diagnosed or not) in our data. Respondents were asked if they had had unprotected anal sex with each partner during the recall periods (8, 18). Predictors included the type of relationship, the HIV diagnosis status of the HIV+ partner (i.e., whether or not he himself knew that he was HIV+), and the disclosure status of the HIV+ partner (whether he had told his partner he was HIV+). Predictors that dropped out of the model included sexual position and perceived monogamy of the partnership.

Base model coefficients for the nine race/partnership types among the adult MSM were defined as logit(P(condom use|anal intercourse) =

|  | **Coefficient** |
| --- | --- |
| Main partnership | -1.325 |
| Casual partnership | -1.046 |
| One-time contact | -1.008 |

Note that for these, the reference category is the case in which the HIV+ man is undiagnosed, hence the relatively low values of condom use. Modifiers for these logit coefficients are:

| **Condition** | **Coefficient** |
| --- | --- |
| HIV+ diagnosis | 0.670 |
| HIV+ status disclosure | 0.850 |

Together, these values, in combination with the frequencies with which AI occurs in all of the different types of situations, implies an overall rate of condom use of approximately 50% across all acts.

For the ASMM relationships the rate of condom use was 54% based on reports of unprotected AI over a 6-week period among those reporting any AI in Hidalgo et al 2015 (20). This was found to be highly consistent with reports from multiple other sources (21-23). The condom use rate applied to any relationship involving an ASMM; no fixed proportion of ASMM always used condoms. Condom use among ASMM was one of the parameters adjusted using Approximate Bayesian Computation to fit the HIV prevalence value chosen as our calibration parameter.

## 4.4 Sexual role

Men are assigned an individual sexual role preference (exclusively insertive, exclusively receptive, or versatile) as described in Section 3.1.6. Relationships between two exclusively insertive or two exclusively receptive men are prohibited via the ERGM and STERGM models. Versatile men are further assigned an insertivity preference drawn from a uniform distribution between 0 and 1. When two versatile men are determined to have an AI act, their sexual positions must be determined (all other combinations have only one feasible combination). One option is for men to engage in intra-event versatility (IEV; i.e., both engage in insertive and receptive AI during the act). The probability of this is 49%, and is derived from the partner-specific role data described in Section 3.1.6. If IEV does not occur, then each man’s probability of being the insertive partner equals his insertivity quotient divided by the sum of the two men’s insertivity quotients.

# 5 DEMOGRAPHY

In this model, there are three demographic processes: entries, exits, and aging.

## 5.1 Entry and sexual debut

All persons enter the network at age 13. The number of new entries at each time step is based on a fixed rate (3 per 10,000 persons per weekly time step) that keeps the overall network size in a stable state over the time series of the simulations. The model parameter governing this rate was calibrated iteratively in order to generate simulations with a population size at equilibrium, given the inherent variability in population flows related to background mortality, sexual maturation (i.e., reaching the upper age limit of 40), and disease-induced mortality. At each time step, the exact number of ASMM entering the population was simulated by drawing from a Poisson distribution with the rate parameter.

At entry each individual is assigned an age (13-18) at which they will become eligible to experience anal intercourse (AI). This is because our population definition consists of those who have not yet self-identified as ASMM, those that have begun to identify as gay/bisexual, and those who identify as ASMM and have begun sexual activity with males. That is, an individual need not have sexually debuted to fall within our population definition.

The probability of each age for self-identifying as an ASMM:

| **Age** | **Probability** |
| --- | --- |
| **13** | **44%** |
| **14** | **13%** |
| **15** | **12%** |
| **16** | **11%** |
| **17** | **10%** |
| **18** | **10%** |

These numbers were derived through back-calculation from the size of the respondent pool in the Youth Risk Behavioral Surveillance System, or YRBSS (24), which began with:

| **Current age** | **Weighted # ASMM** | **Weighted # males** | **Est. % ASMM** |
| --- | --- | --- | --- |
| **14** | **6,648** | **141,975** | **4.7%** |
| **15** | **14,789** | **310,381** | **4.8%** |
| **16** | **20,566** | **309,489** | **6.6%** |
| **17** | **17,957** | **280,313** | **6.4%** |
| **18+** | **14,658** | **181,363** | **8.1%** |

We excluded ages below 14 since the numbers of adolescent sexual minority males (ASMM) were small and the estimates highly variable. We then fit a logarithmic curve to these five numbers:

We selected a logarithmic curve over a linear one since the former recognizes that the proportion of ASMM should asymptote at some older age. We used the resulting equation to develop predicted proportions for each age (13-18) in our model. We then converted these into the proportions identifying as ASMM in our model at each age listed above.

For becoming eligible for sexual activity, we then derive two parameters: one for the probability of becoming eligible for sexual activity at the same time as self-identifying as an ASMM (i.e., first meeting the definition of ASMM because of sexual activity) and, for those ASMM who put off sexual debut, a weekly probability of debuting thereafter.

We then used the data from the same adolescents about their reported age of first intercourse, given their current age:

|  | **AI debut age** | | | | | | |
| --- | --- | --- | --- | --- | --- | --- | --- |
| **Current age** | **13** | **14** | **15** | **16** | **17** | **18** | **No AI** |
| **13** | **65%** |  |  |  |  |  | **35%** |
| **14** | **39%** | **14%** |  |  |  |  | **47%** |
| **15** | **32%** | **15%** | **10%** |  |  |  | **43%** |
| **16** | **32%** | **13%** | **13%** | **11%** |  |  | **31%** |
| **17** | **27%** | **11%** | **13%** | **14%** | **6%** |  | **29%** |
| **18** | **33%** | **10%** | **11%** | **15%** | **17%** | **0%** | **14%** |

We assume, given the lack of data specific to anal intercourse, that for most of these ASMM the age of first intercourse is the age of first anal intercourse. We used Approximate Bayesian computation (ABC) to find the values of our two parameters that, when simulated with the selected rates above, would come closest to generating this matrix of retrospective debut times in a population interviewed at these ages. We used the Beaumont method for sequential ABC, as implemented in the R package EasyABC, with uniform priors on the range (0,1) and (0, 0.002) for our two parameters, and tolerance levels 1000, 100, and 75. We used the mean of all non-rejected parameters values as our final estimate, yielding a probability of AI debut at the age of self-identifying as an ASMM of 50.6%, and a weekly probability of debut thereafter at 0.34%. All individuals were sexually activity at age 19.

## 5.2 Initialization of Attributes

Persons entering the population were assigned attributes, some of which remained fixed by definition (e.g., race), others fixed by assumption (e.g., insertive versus receptive sexual role), and yet others allowed to vary over time (e.g., age and disease status). Here we describe three attributes in the first category:

- For **race/ethnicity**, this model was based on a population composition that was 50% black MSM and 50% white MSM. As noted, we did not explicitly model race within this study, and set all race-specific parameters to averages across stratified estimates. Subsequent models will extend this model framework to explore racial disparities related to PrEP uptake among MSM. This 1:1 ratio comes close to that for the Atlanta metropolitan area and also provides analytical clarity.
- **Circumcision** status was randomly assigned to incoming men. Based on empirical data from Atlanta MSM (18), 89.6% of men were circumcised before sexual onset. Circumcision was associated with a 60% reduction in the per-act probability of infection for HIV- males for insertive anal intercourse only (i.e., circumcision did not lower the *transmission* probability if the HIV+ partner was insertive) (2, 25)
- The **CCR5-Δ32 genetic allele** was modeled by assigning a mutation for zero, one, or two chromosomes. Compared to men without a CCR5 mutation, heterozygous men (those with one mutation) were 70% less likely to become infected and homozygous men (those with two mutations) were fully immune from infection (26, 27). The population distribution of CCR5 was differential by race, with 0% of black men and 3.4% of white men expressing as homozygous, and 2.1% of black men and 17.6% of white men expressing as heterozygous (26). But because race was not explicitly represented in these models, we averaged each set of proportions: 1.7% homozygous and 9.9% heterozygous overall.

## 5.3 Exits from the Network

All persons exited the network by age 40, either from mortality or reaching the upper age bound of the MSM target population of interest. This upper limit of 40 was modeled deterministically (probability = 1), but other exits due to mortality were modeled stochastically. Mortality included both natural (non-HIV) and disease-induced mortality causes before age 40. Background mortality rates were based on US all-cause mortality rates specific to age and race from the National Vital Statistics life tables (28). The following table shows the probability of mortality per year by age and race.

| **Age** | **White** | **Black** |
| --- | --- | --- |
| 13–24 | 0.00103 | 0.00159 |
| 25–34 | 0.00133 | 0.00225 |
| 35–39 | 0.00214 | 0.00348 |

Natural mortality was applied to persons within the population at each time step stochastically by drawing from a binomial distribution for each eligible person with a probability parameter corresponding to that person’s risk of death tied to his age. Disease-related mortality, in contrast, was modeled based on clinical disease progression, as described in Section 6.

## 5.4 Aging

The aging process in the population was linear by time step for all active persons. The unit of time step in these simulations was one week, and therefore, persons were aged in weekly steps between the minimum and maximum ages allow (13 and 40 years old). Evolving age impacted background mortality, age-based mixing in forming new partnerships, and other behavioral features of the epidemic model described below. Persons who exited the network were no longer active and their attributes such as age were no longer updated.

# 6 INTRAHOST EPIDEMIOLOGY

Intrahost epidemiology includes features related to the natural disease progression within HIV+ persons in the absence of clinical intervention. The main component of progression that was explicitly modeled for this study was HIV viral load. In contrast to other modeling studies that model both CD4 and viral load, our study used viral load progression to control both interhost epidemiology (HIV transmission rates) and disease progression eventually leading to mortality.

Following prior approaches (1, 2, 5, 6, 13), we modeled changes in HIV viral load to account for the heighted viremia during acute-stage infection, viral set point during the long chronic stage of infection, and subsequent rise of VL at clinical AIDS towards disease-related mortality. A starting viral load of 0 is assigned to all persons upon infection. From there, the natural viral load curve is fit with the following parameters. The HIV viral load has a crucial impact on the rates of HIV transmission within serodiscordant couples in the model, and this interaction is detailed in Section 8. The parameters governing these processes are provided in the table below.

| **Parameter** | **Value** | **Reference** |
| --- | --- | --- |
| Time to peak viremia in acute stage | 45 days | Little (29) |
| Level of peak viremia | 6.886 log_10_ | Little (29) |
| Time from peak viremia to viral set point | 45 days | Little (29), Leynaert (30) |
| Level of viral set point | 4.5 log_10_ | Little (29) |
| Duration of chronic stage infection (no ART) | 3550 days | Buchbinder (31), Katz (32) |
| Duration of AIDS stage | 728 days | Buchbinder (31) |
| Peak viral load during AIDS (at death) | 7 log_10_ | Estimated from average duration of AIDS |

After infection, it takes 45 days to reach peak viremia, at a level of 6.886 log 10. From peak viremia, it takes another 45 days to reach viral set point, which is set at a level of 4.5 log 10. The total time of acute stage infection is therefore 3 months. The duration of chronic stage infection in the absence of clinical intervention is 3550 days, or 9.7 years. The total duration of pre-AIDS disease from infection is therefore approximately 10 years. At onset of AIDS, HIV viral load rises linearly from 4.5 log 10 to 7 log 10, at which point mortality is assumed to occur. The time spent in the AIDS stage is 728 days, or 2 years. This viral load trajectory is for ART-naïve persons only, and the influence of ART on disease progression is detailed in Section 6. These transitions are deterministic for all ART-naïve persons.

# 7 CLINICAL EPIDEMIOLOGY

Clinical epidemiological processes refer to all steps along the HIV care continuum after initial infection: diagnosis, linkage to care, treatment initiation and adherence, and HIV viral load suppression. In this model, these clinical features have critical interactions with behavioral features detailed above, as well as impacts on the rates of HIV transmission, detailed below. The features of our model’s clinical processes generally follow the steps of the HIV care continuum, in which persons transition across states from infection to diagnosis to medical care linkage and ART initiation to HIV viral suppression.^20^

## 7.1 HIV Diagnostic Testing

Persons in our models were divided into non-testers (through age 40) and regular interval-based testers. Based on empirical data for Atlanta MSM (18), 6.5% of MSM did not receive HIV testing before age 40. This was calculated based on a survey about never tested prior to the study, which may overestimate the final proportion who would have never tested before age 40. A fixed individual attribute for HIV treatment trajectories that characterized progression through the care continuum was randomly assigned upon entry into the population, with this group of 6.5% of MSM not accessing HIV testing or other forms of post-diagnostic HIV medical services.

The remaining 93.5% who entered the HIV care continuum HIV tested at regular intervals. From age 19 through 39, individuals tested at regular intervals with the estimated mean time between tests for HIV-negative persons at 301 days for black MSM and 315 days for white MSM (18) (33). This was calculated based on time since last test in the survey, with the assumption that testing was a memoryless process. In this paper, we averaged over the two intervals since we did not explicitly model racial differences in the care continuum. From age 13 through 18, testing was less frequent. Sharma et al. (24) explore HIV testing among ASMM. In an additional unpublished analysis for this model, they found that 29.2% of sexually experienced 18-year-old ASMM had tested for HIV at least once in their lifetime. We simulated multiple weekly testing hazards until obtaining a value that yielded this outcome. This final selection was for a constant weekly hazard for testing of 0.00238, which corresponds to a mean test interval of 2,900 days.

Diagnostic testing was simulated stochastically using draws from a binomial distribution with probability parameters equal to the reciprocal of the interval for each of the two age groups. This generated a population-level geometric distribution of times since last test within the two groups.

We also modeled a 21-day window period after infection during which the tests of the truly HIV+ persons would show as negative to account for the lack of antibody response immediately after infection (34). HIV+ persons who tested after this window period would be correctly diagnosed with 100% test sensitivity. Individual-level attributes for diagnosis status and time since last HIV test were recorded for all MSM.

## 7.2 Antiretroviral Therapy (ART) Initiation

Consistent with previous models (1, 2, 5, 6, 13), we simulated the initiation of ART and subsequent clinical outcomes of full or partial HIV viral suppression based on men being in one of three clinical states: never tested, on treatment and partially virally suppressed, and on treatment with full viral suppression. There was insufficient empirical data to represent the patterns and rates at which individual men switch among these three states over the course of their infection, since the clinical ART landscape is constantly evolving. Therefore, we modeled men as being on one of the three fixed treatment trajectories as an individual-level attribute such that our model matched the population-level data on the prevalence of durable HIV viral suppression and treatment-naïve mortality (35, 36).

Following HIV diagnosis (for the 93.5% of men who ever HIV test before age 40), MSM initiated treatment at a rate of 0.1095 per week. This translates into an average interval between testing and treatment initiation of 9.13 weeks, consistent with empirical data (33). In the absence of quantitative data, we assumed no gap between treatment entry and ART initiation.

## 7.3 ART Adherence and Viral Suppression

MSM who initiated ART could cycle on and off treatment, where cycling off treatment resulted in an increase in the VL back up to the assumed set point of 4.5 log_10_. The slope of changes to VL were calculated such that it took a total of 3 months to transition between the set point and the on-treatment viral loads (37). Men on treatment could achieve partial or full suppression. Men with partial suppression were assumed to have a log_10_ viral load of 3.5, compared to 1.5 among those who were fully suppressed (37). The latter corresponds to an absolute viral load below the standard levels of detection (VL = 50) (38).

Parameters for treatment initiation, discontinuation, and reinitiation, as well as the proportion of those initiating ART who achieved full suppression were all taken from Goodreau et al. (1), which were in turn estimated from a variety of estimates in the literature(35, 39). Since Goodreau et al, provided race-specific estimates rather than a single set of values overall, we took a weighted average over the race-specific parameter estimates, yielding:

| **Parameter** | |
| --- | --- |
| Proportion of those initiating ART who achieved full suppression | 0.633 |
| Per-week probability of falling out of suppression | 0.0087 |
| Per-week probability of re-achieving suppression | 0.00179 |

## 7.4 Disease Progression and Mortality after ART Initiation

Mortality after ART initiation was modeled based on the cumulative time on and off ART for persons who were fully or partially suppressed. The maximum time between infection and the start of AIDS was 9.7 years (31). A person in either the full or partial suppression categories who spent this much time off ART during the course of infection progressed to AIDS. For the partially suppressed, we assumed a maximum time on ART of 15 years, similar to previous models, to account for treatment failure (1). For this group, the time to AIDS was an additive function of two ratios: (time on treatment / maximum time on treatment) + (time off treatment / maximum time off treatment). AIDS was simulated to occur when the sum of this score exceeded 1. Persons who had ever initiated ART progressed through AIDS at a similar rate as those who were ART-naïve.

# 8 INTERHOST EPIDEMIOLOGY

Interhost epidemiological processes represent the HIV-1 disease transmission within the model. Disease transmission occurs between sexual partners who are active on a given time step. This section will describe the overall rate as a function of the intrahost epidemiological profile of each member of a partnership and behavioral features within the dyad.

## 8.1 Disease-Discordant Dyads

At each time step in the simulation, a list of active dyads was selected based on the current composition of the network. This was called an “edgelist.” Given the three types of partnerships detailed above, the full edgelist was a concatenation of the type-specific sublists. The complete edgelist reflects the work of the STERGM- and ERGM-based network simulations, wherein partnerships formed on the basis of nodal attributes and degree distributions (see Section 2). Dyads were considered active at a specific time step if the terminus of that simulated edge was less than or equal to the current time step (right-censored). From the full edgelist, a disease-discordant subset was created by removing those dyads in which both members were HIV- or both were HIV+. This left dyads that were discordant with respect to HIV status, which was the set of potential partnerships over which infection may be transmitted at that time step.

## 8.2 Per-Act HIV Transmission Probability

Within disease-discordant dyads, HIV transmission was modeled based on a sexual act-by-act basis, in which multiple acts of varying infectiousness could occur within one partnership within a weekly time step. Determination of the number of acts wthin each discordant dyad for the time step, as well as condom use and role for each of those acts, was described in Section 3. Transmission by act was then modeled as a stochastic process for each discordant sex act following a binomial distribution with a probability parameter that is a multiplicative function of the following predictors of the HIV- and HIV+ partners within the dyad.

| **Predictor** | **Partner** | **Parameters** | **References** |
| --- | --- | --- | --- |
| Sexual role (insertive or receptive) | HIV- | *Receptive:* 0.008938 base probability when HIV+ partner has 4.5 log_10_ viral load | Vittinghoff (40) |
|  |  | *Insertive:* 0.003379 base probability when HIV+ partner has 4.5 log_10_ viral load | Vittinghoff (40) |
| HIV viral load (VL) | HIV+ | Multiplier of 2.45^(VL - 4.5)^ | Wilson (41) |
| Acute stage | HIV+ | Multiplier of 6 | Leynaert (30), Bellan (42) |
| CCR5 status | HIV- | Δ32 homozygote: multiplier of 0 | Marmor (26) |
|  |  | heterozygote: multiplier of 0.3 | Marmor (26) |
| Condom use | Both | Multiplier of 0.25 | Varghese (43), Weller (44) |
| Circumcision status | HIV-, insertive | Multiplier of 0.40 | Gray (25) |
| PrEP status | HIV- | Detailed below | – |

For each act, the overall transmission probability was determined first with a base probability that was a function of whether the HIV- partner was in the receptive or insertive role, with the former at a 2.6-fold infection risk compared to the latter. The HIV+ partner’s viral load modifies this base probability in a non-linear formulation, upwards if the VL was above the VL set point during chronic stage infection in the absence of ART, and downwards if it was below the set point. Following others, we modeled an excess transmission risk in the acute stage of infection above that predicted by the heightened VL during that period. Four predictors of the HIV- partner could reduce the risk of infection: the Δ32 allele on the CCRR5 gene, condom use within the act, circumcision status (only if the HIV- partner was insertive in that act), and PrEP status (which we further detail in the following section).

The final transmission rate per partnership per weekly time step was a function of the per-act probability of transmission in each act and the number of acts per time step. The per-act transmission probability could be heterogeneous within a partnership due to various types of acts in each interval: for example, a HIV- man who is versatile in role may have both insertive and receptive intercourse within a single partnership; some acts within a partnership may be protected by condom use while others are condomless. Transmission was simulated for each act within each serodiscordant dyad, based on draws from a binomial distribution with the probability parameter equal to the per-act transmission probabilities detailed above.

# 9 PRE-EXPOSURE PROPHYLAXIS (PrEP)

PrEP was modeled as daily oral use of combination tenofovir disoproxil fumarate and emtricitabine (trade name: Truvada) among HIV- MSM (45). Active PrEP use reduces the per-act probability infection for HIV- men based on the level of adherence to PrEP after initiation. In this section, we further describe the methods for modeling PrEP uptake based on the indications for prescription from CDC’s guidelines for clinical practice, the role of PrEP uptake and monitoring, variable levels of adherence and its impact on HIV susceptibility, and the calculation of the epidemiological outcomes presented in the main paper.

## 9.1 PrEP Indications

For the adult MSM 19-39 years of age, the indications for PrEP initiation followed the eligibility guidelines for prescription within CDC’s recommendations for clinical practice (46). This paper explicitly models only the behavioral components of the PrEP indications for MSM:

1. UAI in monogamous partnerships with a partner not recently tested negative for HIV;
2. UAI outside of a monogamous partnership; and
3. AI in a known serodiscordant partnership.

We modeled PrEP indications based on a “clinical” interpretation of these guidelines that may be more realistic to assess in practice (5).

Following previous work (6) (13), PrEP indications for ASMM were simplified. Any sexually active ASMM older than 16 was eligible for PrEP and initiation began 6 months following eligibility to reflect annual doctor visits.

## 9.2 PrEP Uptake and Monitoring

In our models, diagnostic testing is the gateway through which PrEP is offered. A small percentage of MSM (6.5%) never test before age 40, but the remainder test at regular intervals (approximately yearly before PrEP). Adult MSM are assessed for PrEP indications only at visits in which their HIV test result is negative. At that time, adult MSM are considered for PrEP initiation only if the proportion of men on PrEP has not surpassed a threshold coverage fraction of 40%. Once men initiate PrEP they return to diagnostic testing visits at quarterly intervals. Newly infected men are discontinued on PrEP immediately. On a yearly basis (after 4 quarters of testing after PrEP initiation), their risk behavior is reassessed; if formerly indicated adult MSM had no behavioral indications in the window period before that reevaluation, their PrEP is discontinued.

For the ASMM, any sexually active HIV- individual at least 16 years of age is eligible for PrEP. Once an ASMM is eligibility for PrEP they begin PrEP use after six months reflecting the average time between annual check-ups. ASMM will only begin PrEP if the number of ASMM on PrEP has not surpassed a threshold coverage fraction which we vary from 10% to 30%. Based on preliminary data from ATN113 (47), we then set a constant weekly probability of discontinuation (1.43%) such that 50% of those initiating had discontinued by Week 48. Newly infected ASMM are discontinued on PrEP immediately.

## 9.3 Adherence and Impact on HIV Transmission

Individuals initiating PrEP were assigned a fixed adherence profile that reflected an average weekly dosage. Adherence parameters for adult MSM were drawn from an open-label demonstration project reweighted by race to account for the small proportion of non-white persons in that study (48). Our model assigned 21.1% of men as non-adherent, 7.0% as taking <2 pills/week, 10.0% 2–3 pills/week, and 61.9% at 4+ pills/week. For the ASMM, we derived our levels of the four adherence categories among ASMM from the preliminary data reported from the ATN113 trial (47), averaged across each of the follow-up timepoints, yielding 20.9%, 24.4%, 13.1% and 41.6% of ASMM in each adherence category, from low to high. For both groups, use of PrEP resulted in a reduction of the per-act probability of infection correlated with adherence level: 0%, 31%, 81%, and 95%, for the non-adherent to high-adherence groups, respectively, following Grant et al. (45).

# 10 SIMULATION METHODS

This section describes the methods for executing the simulations and conducting the data analysis on the outcomes in further detail.

## 10.1 Model Calibration

We replicate the current HIV epidemic in this population using model parameters measured recently following existing approaches (1, 2, 5, 6, 13). Starting with a population of 13,500 MSM, HIV infection was initially seeded in 28% of the adult population and 7% of the ASMM population. A set of burn-in simulations was then used to allow the natural dynamics of HIV transmission, demography, and other population features to evolve over time. The goal of the burn-in simulation was to arrive at a network of MSM that was independent of the initial conditions resulting from the seeding. This also established a population composition with behavioral and biological features calibrated to match the Atlanta-area HIV prevalence of 26% among the adult MSM (18, 49) and 7% among 18-year-old ASMM.

We used Approximate Bayesian computation with sequential Monte Carlo sampling (ABC-SMC) methods (42, 50) to calibrate behavioral parameters in which there was measurement uncertainty in order to match the simulated HIV prevalence at the end of the burn-in simulations to the HIV prevalence among both the adults and 18-year-old ASMM.

In this use of ABC, the parameters to be calibrated were overall multipliers for the rate of acts within the adult persistent partnerships over time and the ASMM partnerships over time as well as the probability of condom use within ASMM partnerships. We chose these parameters based on the assumption that they all may be subject to sensitivity biases. We used a uniform prior distribution for the multiplier parameters, with a range from 1 to 10, where 1 was equal to the act rate observed in the data. The prior distribution on the probability of using a condom within ASMM partnerships ranged from 0 to 1.

For summary statistics against which to measure the performance of the model simulations, we choose two for this research project: 1) the prevalence value at the end of the time series for the adult MSM; and 2) the prevalence value at the end of the time series for ASMM age 18. The ABC model calibration was completed using the EasyABC package in R.

For the ABC algorithms to calibrate to the observed HIV prevalence, a total of 480 simulations were required for 50 years of calendar time. The posterior distribution produced a mean 16.9% daily probability of AI within casual relationships among adult MSM, a 24.1% daily probability of AI within an ASMM relationship and a 23% probability that an ASMM would use a condom during AI. Given this parameter set, we simulated a burn-in model 100 times to account for the stochastic variability over individual simulations. The individual simulation in which the HIV prevalence at the end of the simulation was closest to the HIV prevalence target statistics was selected as the starting point for the intervention simulations.

## 10.2 Intervention Simulations

The intervention scenarios are described fully within the main paper. For each scenario, we simulated the model scenario 100 times for 40 calendar years in each simulation. Data from each simulation were merged, and a complete 100-simulation data file was retained for each scenario. All burn-in and intervention simulations were conducted on the Hyak high-performance computing platform at the University of Washington.

# 11 REFERENCES

1. Goodreau SM, Carnegie NB, Vittinghoff E, Lama JR, Sanchez J, Grinsztejn B, et al. What drives the US and Peruvian HIV epidemics in men who have sex with men (MSM)? PLoS One. 2012;7(11):e50522.

2. Goodreau SM, Carnegie NB, Vittinghoff E, Lama JR, Fuchs JD, Sanchez J, et al. Can male circumcision have an impact on the HIV epidemic in men who have sex with men? PLoS One. 2014;9(7):e102960.

3. Carnegie NB, Goodreau SM, Liu A, Vittinghoff E, Sanchez J, Lama JR, et al. Targeting pre-exposure prophylaxis among men who have sex with men in the United States and Peru: partnership types, contact rates, and sexual role. Journal of acquired immune deficiency syndromes (1999). 2015;69(1):119-25.

4. Goodreau SM, Rosenberg ES, Jenness SM, Luisi N, Stansfield SE, Millett G, et al. Sources of racial disparities in HIV prevalence among men who have sex with men in Atlanta, GA: A modeling study. Lancet HIV. 2017;4(7):e311-e20.

5. Jenness SM, Goodreau SM, Rosenberg E, Beylerian EN, Hoover KW, Smith DK, et al. Impact of the Centers for Disease Control's HIV pre-exposure prophylaxis guidelines for men who have sex with men in the United States. J Infect Dis. 2016.

6. Goodreau SM, Hamilton DT, Jenness S, Sullivan P, Valencia R, Wang LY, et al. Targeting HIV pre-exposure prophylaxis to adolescent sexual minority males in higher prevalence areas of the United States: a modeling study. 2017.

7. Handcock MS, Hunter DR, Butts CT, Goodreau SM, Morris M. statnet: Software tools for the representation, visualization, analysis and simulation of network data. J Stat Softw. 2008;24(1).

8. Grey JA, Rothenberg RB, Sullivan PS, Rosenberg ES. Disassortative age-mixing does not explain differences in HIV prevalence between young White and Black MSM: Findings from four studies. PLoS One. 2015;10(6):e0129877.

9. Whiteside YO, Song R, Wertheim JO, Oster AM. Molecular analysis allows inference into HIV transmission among young men who have sex with men in the United States. AIDS. 2015;29(18):2517-22.

10. Halkitis PN, Kapadia F, Siconolfi DE, Moeller RW, Figueroa RP, Barton SC, et al. Individual, psychosocial, and social correlates of unprotected anal intercourse in a new generation of young men who have sex with men in New York City. Am J Public Health. 2013;103(5):889-95.

11. Sanchez T, Zlotorzynska M, Sineath C, Kahle E, Sullivan P. The annual American Men's internet survey of behaviors of men who have sex with men in the United States: 2014 key indicators report. JMIR Public Health Surveill. 2016;2(1):e23.

12. Sullivan PS, Carballo-Diéguez A, Coates T, Goodreau SM, McGowan I, Sanders EJ, et al. Successes and challenges of HIV prevention in men who have sex with men. The Lancet. 2012;380(9839):388-99.

13. Hamilton DT, Goodreau SM, Jenness SM, Sullivan PS, Wang LY, Dunville RL, et al. Potential impact of HIV pre-exposure prophylaxis among black and white adolescent sexual minority males. AJPH. 2018;108(S4).

14. Krivitsky PN, Handcock MS. A separable model for dynamic networks. J R Stat Soc B. 2014;76(1):29-46.

15. Hunter DR, Handcock MS, Butts CT, Goodreau SM, Morris M. ergm: A package to fit, simulate and diagnose exponential-family models for networks. J Stat Softw. 2008;24(3):nihpa54860.

16. Carnegie NB, Krivitsky PN, Hunter DR, Goodreau SM. An approximation method for improving dynamic network model fitting. J Comput Graph Stat. 2015;24(2):502-19.

17. Krivitsky PN, Handcock MS, Morris M. Adjusting for network size and composition effects in exponential-family random graph models. Stat Methodol. 2011;8(4):319-39.

18. Sullivan PS, Rosenberg ES, Sanchez TH, Kelley CF, Luisi N, Cooper HL, et al. Explaining racial disparities in HIV incidence in black and white men who have sex with men in Atlanta, GA: a prospective observational cohort study. Annals of epidemiology. 2015;25(6):445-54.

19. Rotheram-Borus MJ, Reid H, Rosario M. Factors mediating changes in sexual HIV risk behaviors among gay and bisexual male adolescents. American Journal of Public Health. 1994;84(12):1938-46.

20. Hidalgo MA, Kuhns LM, Hotton AL, Johnson AK, Mustanski B, Garofalo R. The MyPEEPS randomized controlled trial: a pilot of preliminary efficacy, feasibility, and acceptability of a group-level, HIV risk reduction intervention for young men who have sex with men. Arch Sex Behav. 2015;44(2):475-85.

21. Sifakis F, Hylton JB, Flynn C, Solomon L, Mackellar DA, Valleroy LA, et al. Racial disparities in HIV incidence among young men who have sex with men: the Baltimore Young Men's Survey. Journal of acquired immune deficiency syndromes (1999). 2007;46(3):343-8.

22. Pathela P, Schillinger JA. Sexual behaviors and sexual violence: adolescents with opposite-, same-, or both-sex partners. Pediatrics. 2010;126(5):879-86.

23. Everett BG, Schnarrs PW, Rosario M, Garofalo R, Mustanski B. Sexual orientation disparities in sexually transmitted infection risk behaviors and risk determinants among sexually active adolescent males: results from a school-based sample. Am J Public Health. 2014;104(6):1107-12.

24. Sharma A, Wang LY, Dunville R, Kearns R, Rosenberg E, Sullivan P. HIV and STD testing behavior among young men who have sex with men: Analysis of pooled Youth Risk Behavior Survey data, 2005-2013. Journal of Adolescent Health. 2016;58(2):S80.

25. Gray RH, Kigozi G, Serwadda D, Makumbi F, Watya S, Nalugoda F, et al. Male circumcision for HIV prevention in men in Rakai, Uganda: a randomised trial. The Lancet. 2007;369(9562):657-66.

26. Marmor M, Sheppard HW, Donnell D, Bozeman S, Celum C, Buchbinder S, et al. Homozygous and heterozygous CCR5-Delta32 genotypes are associated with resistance to HIV infection. Journal of acquired immune deficiency syndromes (1999). 2001;27(5):472-81.

27. Zimmerman PA, Buckler-White A, Alkhatib G, Spalding T, Kubofcik J, Combadiere C, et al. Inherited resistance to HIV-1 conferred by an inactivating mutation in CC chemokine receptor 5: studies in populations with contrasting clinical phenotypes, defined racial background, and quantified risk. Mol Med. 1997;3(1):23-36.

28. United States Census Bureau. Mortality Data. In: Bureau USC, editor. 2012.

29. Little SJ, McLean AR, Spina CA, Richman DD, Havlir DV. Viral dynamics of acute HIV-1 infection. J Exp Med. 1999;190(6):841-50.

30. Leynaert B, Downs AM, de Vincenzi I. Heterosexual transmission of human immunodeficiency virus: variability of infectivity throughout the course of infection. European Study Group on Heterosexual Transmission of HIV. Am J Epidemiol. 1998;148(1):88-96.

31. Buchbinder SP, Katz MH, Hessol NA, O’Malley PM, Holmberg SD. Long-term HIV-1 infection without immunologic progression. AIDS. AIDS. 1994;8(8):1123-8.

32. Katz MH, Hessol NA, Buchbinder SP, Hirozawa A, O'Malley P, Holmberg SD. Temporal trends of opportunistic infections and malignancies in homosexual men with AIDS. J Infect Dis. 1994;170(1):198-202.

33. Rosenberg ES, Millett GA, Sullivan PS, del Rio C, Curran JW. Understanding the HIV disparities between black and white men who have sex with men in the USA using the HIV care continuum: a modelling study. The Lancet HIV. 2014;1(3):e112-e8.

34. Fiebig EW, Wright DJ, Rawal BD, Garrett PE, Schumacher RT, Peddada L, et al. Dynamics of HIV viremia and antibody seroconversion in plasma donors: implications for diagnosis and staging of primary HIV infection. AIDS. 2003;17(13):1871-9.

35. Beer L, Oster AM, Mattson CL, Skarbinski J, Medical Monitoring P. Disparities in HIV transmission risk among HIV-infected black and white men who have sex with men, United States, 2009. AIDS. 2014;28(1):105-14.

36. Bertolli J, Shouse RL, Beer L, Valverde E, Fagan J, Jenness SM, et al. Using HIV surveillance data to monitor missed opportunities for linkage and engagement in HIV medical care. Open AIDS J. 2012;6:131-41.

37. Chu H, Gange SJ, Li X, Hoover DR, Liu C, Chmiel JS, et al. The effect of HAART on HIV RNA trajectory among treatment-naive men and women: a segmental Bernoulli/lognormal random effects model with left censoring. Epidemiology. 2010;21 Suppl 4:S25-34.

38. Chun TW, Carruth L, Finzi D, Shen X, DiGiuseppe JA, Taylor H, et al. Quantification of latent tissue reservoirs and total body viral load in HIV-1 infection. Nature. 1997;387(6629):183-8.

39. Hall HI, Frazier EL, Rhodes P, Holtgrave DR, Furlow-Parmley C, Tang T, et al. Differences in human immunodeficiency virus care and treatment among subpopulations in the United States. JAMA Intern Med. 2013;173(14):1337-44.

40. Vittinghoff E, Douglas J, Judson F, McKirnan D, MacQueen K, Buchbinder SP. Per-contact risk of human immunodeficiency virus transmission between male sexual partners. Am J Epidemiol. 1999;150(3):306-11.

41. Wilson DP, Law MG, Grulich AE, Cooper DA, Kaldor JM. Relation between HIV viral load and infectiousness: a model-based analysis. The Lancet. 2008;372(9635):314-20.

42. Bellan SE, Dushoff J, Galvani AP, Meyers LA. Reassessment of HIV-1 acute phase infectivity: accounting for heterogeneity and study design with simulated cohorts. PLoS Med. 2015;12(3):e1001801.

43. Varghese B, Maher JE, Peterman TA, Branson BM, Steketee RW. Reducing the risk of sexual HIV transmission: quantifying the per-act risk for HIV on the basis of choice of partner, sex act, and condom use. Sex Transm Dis. 2002;29(1):38-43.

44. Weller SC, Davis-Beaty K. Condom effectiveness in reducing heterosexual HIV transmission. Cochrane Database of Systematic Reviews. 2002.

45. Grant RM, Anderson PL, McMahan V, Liu A, Amico KR, Mehrotra M, et al. Uptake of pre-exposure prophylaxis, sexual practices, and HIV incidence in men and transgender women who have sex with men: a cohort study. Lancet Infect Dis. 2014;14(9):820-9.

46. Cenetrs for Disease Control and Prevention. Preexposure prophylaxis for the prevention of HIV infection in the United States-2014: a clinical practice guideline. vol 6 Atlanta: U.S. Public Health Service 2014 [Available from: <http://www.cdc.gov/hiv/pdf/guidelines/PrEPguidelines2014.pdf>.

47. Hosek SG, Landovitz RJ, Kapogiannis B, Siberry GK, Rudy B, Rutledge B, et al. Safety and feasibility of antiretroviral preexposure prophylaxis for adolescent men who have sex with men aged 15 to 17 years in the United States. JAMA Pediatr. 2017;171(11):1063-71.

48. Liu AY, Cohen SE, Vittinghoff E, Anderson PL, Doblecki-Lewis S, Bacon O, et al. Preexposure prophylaxis for HIV infection integrated with municipal- and community-based sexual health services. JAMA Intern Med. 2016;176(1):75-84.

49. Hernandez-Romieu AC, Sullivan PS, Rothenberg R, Grey J, Luisi N, Kelley CF, et al. Heterogeneity of HIV prevalence among the sexual networks of Black and White men who have sex with men in Atlanta: Illuminating a mechanism for increased HIV risk for young Black men who have sex with men. Sex Transm Dis. 2015;42(9):505-12.

50. Toni T, Welch D, Strelkowa N, Ipsen A, Stumpf MPH. Approximate Bayesian computation scheme for parameter inference and model selection in dynamical systems. Journal of The Royal Society Interface. 2009;6(31):187-202.
